# PARP1-Driven Apoptosis in Chronic Lymphocytic Leukemia

**DOI:** 10.1155/2014/106713

**Published:** 2014-08-03

**Authors:** Panagiotis T. Diamantopoulos, Maria Sofotasiou, Vasiliki Papadopoulou, Katerina Polonyfi, Theodoros Iliakis, Nora-Athina Viniou

**Affiliations:** Department of Internal Medicine, Hematology Unit, Laikon General Hospital, National and Kapodistrian University of Athens, 11527 Athens, Greece

## Abstract

Chronic lymphocytic leukemia (CLL) is considered a malignancy resulting from defects in apoptosis. For this reason, targeting apoptotic pathways in CLL may be valuable for its management. Poly [ADP-ribose] polymerase 1 (PARP1) is the main member of a family of nuclear enzymes that act as DNA damage sensors. Through binding on DNA damaged structures, PARP1 recruits repair enzymes and serves as a survival factor, but if the damage is severe enough, its action may lead the cell to apoptosis through caspase activation, or necrosis. We measured the *PARP1* mRNA and protein pretreatment levels in 26 patients with CLL and the corresponding posttreatment levels in 15 patients after 3 cycles of immunochemotherapy, as well as in 15 healthy blood donors. No difference was found between the pre- and posttreatment levels of PARP1, but we found a statistically significant relative increase of the 89 kDa fragment of PARP1 that is cleaved by caspases in the posttreatment samples, indicating PARP1-related apoptosis in CLL patients after treatment. Our findings constitute an important step in the field, especially in the era of PARP1 inhibitors, and may serve as a base for future clinical trials with these agents in CLL.

## 1. Introduction

The poly [ADP-ribose] polymerases (PARPs) are a family of nuclear enzymes comprising 17 members. Their main function is to bind to DNA breaks, serving as a signal to other DNA-repairing enzymes, in order to fix the damage. Binding of PARPs to DNA leads to their polymerization, and by poly [ADP-ribosylation], a posttranslational modification of proteins playing a crucial role in many cell processes, they participate in DNA repair and gene transcription [1, 2].

Among the members of the PARP family, PARP1 is the most abundant and plays a role in the repair of single-strand DNA (ssDNA) and double-strand DNA (dsDNA) breaks. Inhibition of PARP1 activity leads to reduced ssDNA break repair, eventually leading to cell death. The molecular structure of PARP1 consists of 4 domains, an N-terminal double zinc finger DNA-binding domain, a nuclear localization signal, a central automodification domain, and a C-terminal catalytic domain [3]. PARP1 has a low enzymatic activity, which is stimulated by allosteric activators, such as damaged DNA (single- and double-strand breaks, crossovers, cruciform, and supercoils), undamaged DNA structures, nucleosomes, and some protein-binding partners. Binding of PARP1 with such molecules boosts its enzymatic activity that targets core histones, histone H1 and transcription-related factors [4–8]. Upon binding to these allosteric activators, PARP1 recruits various proteins involved in the DNA damage response to the sites of DNA damage [3], and this means that PARP1 acts essentially as a DNA damage sensor [4]. Low level DNA damage seems to trigger detection and repair of the DNA damage. In that case, PARP1 acts as a survival factor. On the other hand, high levels of DNA damage may lead to cell death by either apoptosis or necrosis through PARP1 overactivation [9].

PARP1 may induce apoptosis, through apoptosis inducing factor (AIF) activation, as well as necrosis. The cell type and the type, strength, and duration of the stimuli are presumed to be factors determining the cell death pathway. It has been shown that actively proliferating cells (such as malignant cells) are more sensitive to PARP1 activation and die by necrosis, while nonproliferating cells are resistant to cell death under the same conditions [10], a fact that is mainly determined by the availability of ATP in the cell [11]. Strong stimuli, such as severe DNA damage, may lead to necrosis through overactivation of PARP1 which causes depletion of the NAD+ and ATP pool of the cell [12, 13].

During the execution phase of apoptosis, caspases cleave several proteins that are necessary for the cell function and survival. Among them, PARP1 is cleaved by caspases 3 and 7 into a ~25 kDa N-terminal fragment containing the DNA-binding domain (DBD) and a ~85 kDa C-terminal fragment that retains basal enzymatic activity but cannot be stimulated by DNA damage [14]. This cleavage is necessary to eliminate PARP1 activation in response to DNA fragmentation, protecting the cells from ATP depletion and subsequent necrotic death, and preventing futile attempts of DNA repair. Through these processes, PARP1 cleavage may help to commit cells to the apoptotic pathway [15]. Thus, PARP1 plays a central role in apoptosis determining the cell fate [16].

CLL is a highly heterogeneous disease in terms of biology and hence clinical presentation. The clinical course of CLL can vary from asymptomatic and indolent for several years to severely symptomatic since diagnosis, requiring treatment. Clinical staging, age, and performance status remain the major factors defining prognosis and need for treatment. New prognostic factors include cytogenetic analysis, immunoglobulin mutation analysis, and expression of 70 kDa zeta associated protein (ZAP-70) and CD38 [17, 18]. Several studies have identified the signal transduction pathways that contribute to antiapoptotic signaling in CLL cells, and CLL is considered a malignancy resulting from defects in apoptosis [19].

Among other genetic defects, defects in the ds-DNA break response have been implicated in the pathogenesis of CLL. Impairment of the DNA damage response has been correlated to aggressive CLL [20], unresponsiveness to standard therapy, and adverse clinical outcomes of patients with CLL [21].

A recent study showed that reduced expression of PARP1 is associated with an impairment of CLL responsiveness to cell death [22]. This is, to our knowledge, the only study on PARP1 expression in CLL.

As PARP1 inhibitors are currently under study in the context of phase II [23, 24] and phase III clinical trials [25], mostly for advanced or relapsed breast and ovarian cancer, the need to further understand the role of PARP1 in hematological malignancies is mandatory. This study tries to shed light on the possible role of PARP1 in the pathways that drive apoptosis in CLL. The aim of our study is to determine the levels of PARP1 expression in patients with CLL before and after immunochemotherapy as well as to compare them with those of healthy individuals.

## 2. Patients and Methods

### 2.1. Patients

Twenty-six patients with B-cell chronic lymphocytic leukemia (CLL) were included in the study. Informed consent was obtained from all patients. The diagnosis of CLL was established in each case using morphological, histopathological, and immunophenotypic criteria. All patients had immunophenotypically confirmed disease by peripheral blood at the time of first sample collection. Fifteen patients among them received treatment with rituximab based immunochemotherapy according to common clinical practice after the first sample collection, and a second sample was obtained after 3 treatment cycles. We also obtained blood samples from 15 healthy blood donors, to be used as a control group.

We obtained from all patients and healthy controls peripheral whole blood samples that were collected in ethylenediaminetetraacetic acid (EDTA). All samples were processed within 6 hours from collection. Following RNA extraction and cDNA synthesis, the samples were kept at −80°C. A quantitative real-time polymerase chain reaction (qRT-PCR) was performed in order to measure* PARP1* mRNA levels. Moreover, PARP1 protein was detected by an immunoblotting assay following protein extraction, as described below.

### 2.2. Methods

#### 2.2.1. RNA Extraction and Reverse Transcription

The Trizol protocol (Invitrogen, Carlsbad, CA, USA) was used to extract and purify total RNA from peripheral whole blood samples. Reverse transcription was performed using MMLV-derived reverse transcriptase enzyme (M-MLV RT, Invitrogen), according to standard protocols.

#### 2.2.2. Primer Design for Real-Time PCR

Primers for PARP1 and *β*-actin were designed with the help of the Primer3 software (University of Massachusetts, USA), using the relevant annotated cDNA sequences from NCBI BLAST (NM_001618.3 for PARP1 and NM_001101.3 for *β*-actin)—primer sequences: for PARP1 forward, CCTGATCCCCCACGACTTT; reverse, GCAGGTTGTCAAGCATTTC and for *β*-actin forward, AGGATGCAGAAGGAGATCACT; reverse GGGTGTAACGCAACTAAGTCATAG.

#### 2.2.3. Real-Time PCR

Real-time PCR was performed with the use of 2X iTaq Universal SYBR GREEN Supermix (Bio-Rad, California, USA) on a CFX96 Real-Time PCR system (Bio-Rad, California, USA) using the following cycling conditions for both PARP1 and *β*-actin: 5′′ at 95°C, 15′′ at 59°C, and 5′′ at 72°C, all steps repeated for 40 cycles. Relative quantitation of PARP1 and *β*-actin transcripts was performed with the standard curve method. PARP1 expression was in fact compared between samples as a ratio of PARP1/actin transcript levels.

#### 2.2.4. Immunoblotting

Total cellular protein was obtained from about 107 cells from each sample, using RIPA buffer. Lysates were incubated on ice for 15 minutes and then centrifuged for 10 minutes at 14,000 rpm. Protein extracts were then separated by SDS-PAGE electrophoresis on acrylamide 5% stacking and 7.5% separating gels, using the Mini-Protean electrophoresis cell (BioRad), according to standard procedures. Molecular weight values were estimated using prestained protein markers (Full Range Rainbow Marker, GE Healthcare). Proteins were transferred from the gel to PVDF membrane (Immun-blot, Biorad), according to the manufacturer's instructions. Membranes were then incubated in blocking solution (5% w/v BSA in TBS-T, i.e., Tris-buffered saline/0.1% Tween 20) for 1 hour at room temperature and the primary antibody was added at a dilution 1/1000 (PARP rabbit mAb, Cat. number 9542, Cell Signal, or *β*-actin rabbit polyclonal Ab, Cat. number 4967, Cell Signal, when membranes were reprobed for loading control). After overnight incubation at 4°C, the membrane was washed 3x in TBS-T and incubated with secondary antibody at a dilution 1/4000 in blocking buffer for 1 h at room temperature (anti-rabbit IgG, HRP conjugated, Cat. number 7074, Cell Signal). After 3x washes in TBS-T, signal was detected with ECL Blotting reagent (GE Healthcare) and X-OMAT LS-1 film (Kodak).

### 2.3. Statistical Analysis

For the statistical analysis of the results we used IBM SPSS statistics, version 19.0. The Related Samples Wilcoxon Signed Rank test was used for comparisons involving pre- and posttreatment values, while the Independent Samples Mann-Whitney *U* test was used to compare the levels of* PARP1* mRNA and protein between patients and healthy controls.

## 3. Results

Whole blood samples were obtained from 26 patients with CLL before treatment and from 15/26 following 3 cycles of immunochemotherapy. Whole blood samples were also obtained from 15 healthy volunteers. The patients' characteristics are shown in Table 1. Data is presented for the total population (26 patients) as well as for the subset of 15 patients from whom samples were obtained both before and after treatment. The vast majority (13/15, 86.6%) of this subset of patients were treatment naïve at the time of first sample collection, while the rest (2/15, 13.3%) had not received any treatment for at least 6 months. None of the above patients had been treated with rituximab in the past. The programmed and eventually administered treatment schemes are shown in Table 1.

The pretreatment levels of* PARP1* mRNA (ratio of* PARP1* to* ACTB* mRNA levels) were found to be 0.088 (0.001–3.490), while the posttreatment value was 0.055 (0.003–0.535). The two values did not differ in a statistically significant level (*P* = 0.51). Moreover, the pretreatment levels of* PARP1* mRNA did not differ significantly from those of the control group (*P* = 0.364), although the control group levels were slightly higher (0.241; range 0.024–1.762).

The used PARP1 antibody detects the endogenous levels of full length PARP1 (116 kDa), as well as the large fragment (89 kDa) of PARP1 resulting from caspase cleavage. We detected the pre- and posttreatment levels of both molecules (full length and large fragment) and we calculated the ratio of their expression (i.e., 116/89). This ratio was used as an indicator of caspase activation. Specifically, a decrease of this ratio would imply a relative increase of the 89 kDa fragment that results from caspase activation in comparison to the full length molecule. On the contrary, an increase of this ratio would mean a relative reduction of the caspase derived fragment.

**Figure 1:**
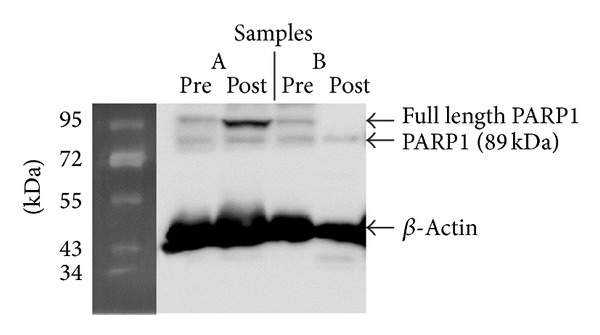
Pre- and posttreatment immunobloting results for 2 patients (A and B). Patient A expresses different levels of the full length (116 kDa) and the 89 kDa fragment of PARP1. Patient B expresses both the full length and the 89 kDa fragment of PARP1 before treatment but loses the full length molecule after immunochemotherapy.

The 89 kDa fragment was detected in all samples (pre- and posttreatment), while the 116 kDa molecule was detected in 22/26 pretreatment samples and in 12/15 posttreatment samples. For these patients, the 116/89 ratio was not calculated, and they were excluded from the statistical analysis. The pre- and posttreatment levels of both the full length molecule and the large fragment of PARP1 did not differ significantly. On the contrary, the pretreatment 116/89 ratio was higher than its posttreatment value (1.245 (0.754–1.589) versus 1.095 (0.444–1.554)), and this difference was statistically significant (*P* = 0.026). The results are presented in detail in Table 2.  Figure 1 shows the immunoblotting results of two patients before and after immunochemotherapy.

The full length molecule of 116 kDa was detected in only one (1/15) of the healthy subjects, while the caspase derived 89 kDa fragment was detected in all of them. The median level of the 89 kDa fragment in the control group was 0.494 (0.172–0.985) and was lower than the pretreatment levels of the patients (*P* = 0.036). Due to the absence of the 116 kDa molecule in the vast majority (14/15) of the healthy controls, the 116/89 ratio was not calculated; thus further correlations were not possible between the control and the patient groups.

Multivariate analysis did not reveal statistically significant differences in the mRNA and protein levels in correlation to the stage of disease, the peripheral blood lymphocyte count, the LDH levels, and the response to treatment. More specifically, there was no statistically significant correlation of the difference of the pre- and posttreatment 116/89 ratios with the response to treatment (*P* = 0.378).

## 4. Discussion

Physiological apoptosis is a process that controls cell numbers, as well as tissue and organ morphology, and removes injured and mutated cells [26]. Dysregulation of apoptotic pathways may result in cancer or other hyperproliferative disorders [27, 28]. The caspases are highly specialized proteases that, when activated, incite one of the more common apoptosis pathways. Upon caspase activation, cell death is initiated through cleavage of several key proteins required for cellular function and survival [29]. Cleavage of PARP1 is considered to be a hallmark of apoptosis [14]. All members of the caspase family may modify PARP1, but caspases 3 and 7 tend to cleave PARP1 in a way that results in the formation of two fragments with specific functions: an 89 kDa catalytic fragment and a 24 kDa DNA binding domain [30]. The 89 kDa fragment has a greatly reduced DNA binding capacity and is released from the nucleus into the cytosol [31]. The 24 kDa fragment binds irreversibly to the DNA strand breaks and inhibits DNA repair enzymes (including PARP1) leading to attenuation of DNA repair [32]. Although the main role of PARP1 is to detect and repair DNA damage, a severe DNA damage could result in high NAD+ and ATP consumption through PARP1 overactivation, leading to depletion of the cell ATP pool. This activity would inevitably lead to necrotic cell death, a process that is blocked by the rapid cleavage and inactivation of PARP1 by the caspases [33, 34]. Thus, when the damage is “too severe to handle” the action of caspases may shift the cell, through enhanced PARP1 cleavage, from necrosis to apoptosis.

We detected, in our samples, the* PARP1* mRNA using a PCR and the corresponding protein (the full length molecule as well as the cleaved by caspases 89 kDa fragment of PARP1), using an immunoblotting technique. By measuring the levels of PARP1 in both RNA and protein levels, we managed to crosscheck our results and most importantly to measure both the “production” and the “usage” of PARP1.

We did not detect any differences in the level of* PARP1* mRNA yield before and after treatment, but we found a statistically significant difference in the ratio of the full length molecule to the 89 kDa fragment before and after immunochemotherapy, indicating caspase activation as reflected by the relatively higher levels of the 89 kDa fragment in the posttreatment samples. Moreover, we found that PARP1 driven apoptosis is probably lower in healthy persons, as indicated by the lower levels of the 89 kDa fragment, in comparison to patients with CLL, a fact that is compatible with the basic speculation that PARP1 driven apoptosis is an indicator of DNA damage which is fundamental in the pathogenesis of CLL and neoplasia in general.

Our results suggest a possible role of PARP1 induced apoptosis in patients with CLL that are treated with rituximab based immunochemotherapy. This preliminary result could serve as a clinical basis for further research in this field and for the use of PARP1 inhibitors in patients with CLL in the context of clinical trials.

Our finding is of significant value for two major reasons. Firstly, it confirms the results of other investigators who measured the levels of PARP1 before and after irradiation treatment of CLL cells [22]. The results of their study indicate that PARP1 is downregulated in nonresponder versus responder samples and that its basal expression is positively correlated with PARP1 cleavage after irradiation. Secondly, our study is the first—to our knowledge—to measure the levels of PARP1 in patient samples before and after “in vivo” treatment administration, and this fact increases the importance of the finding and correlates it more directly to the possible results of the administration of PARP1 inhibitors in CLL.

A drawback of this study is that, due to the rather small study population, no further analysis could be made for the several prognostic factors such as p53 mutation and the immunoglobulin variable (IgVH) region mutation status. Moreover, due to the small number of patients (4/15) treated with fludarabine containing regimens, no correlations of PARP1 expression could be made between patients treated with more or less aggressive regimens.

The molecular mechanisms involved in balancing survival and death of B lymphocytes in CLL triggered by PARP1 activation are highly complex and incompletely understood. According to our results, the regulative action of caspases on PARP1 seems to be important in CLL. We consider this finding of significant value, because it helps to further understand the pathophysiology of the disease and to define the apoptotic pathways that are defective in CLL. Because CLL is considered a malignancy resulting from defects in apoptosis, targeting apoptotic pathways in CLL is a valuable weapon in the treatment of the disease, and our preliminary results could guide future research on whether PARP1 serves as a treatment target in CLL. The extension of this study can provide more detailed information about the role of PARP1 and caspases in several subsets of patients, based on their genetic profile, and could help formulate a plan about the possible use of PARP1 inhibitors in CLL.

## Figures and Tables

**Table 1:** Patient characteristics: epidemiology, disease characteristics, treatment, and response.

Characteristic	All patients	Subset of patients that received treatment
Number of patients, *N* (%)	26 (100)	15 (100)
Median age, years (range)	74 (51–87)	73 (51–82)
Male to female ratio	1.5	1.4
Peripheral blood lymphocytes, ×10^9^/L (range)	29.4 (3.9–81.0)	26.7 (3.9–81.0)
LDH/ULN at presentation, mean (range)	1.2 (0.9–3.1)	1.1 (0.9–2.7)
Previous treatment, *N* (%)	2 (7.7)	2 (13.3)
Disease stage (Binet) (15.24), *N* (%)		
A	10 (38.5)	0 (0)
B	9 (34.6)	8 (53.3)
C	7 (26.9)	7 (46.7)
Immunochemotherapeutic regimen, *N* (%)	NA	
R		8 (53.3)
R, Ch		3 (20.0)
FCR		4 (26.7)
Response to treatment, *N* (%)	NA	
Complete response		3 (20.0)
Partial response		10 (66.7)
Stable disease		2 (13.3)
Disease progression		0 (0)

ULN: upper limit of normal; R: rituximab; Ch: chlorambucil; F: fludarabine; C: cyclophosphamide.

**Table 2:** PCR and immunoblotting results.

	All patients	15 patients (pretreatment)	15 patients (posttreatment)	P*
PARP1-mRNA, median (range)^1^	0.094 (0.001–3.490)	0.088 (0.001–3.490)	0.055 (0.003–0.535)	0.507
116 kDa fragment, median (range)^2^	0.532 (0–1.808)	0.528 (0.263–0.673)	0.551 (0.311–0.864)	0.308
89 kDa fragment, median (range)^2^	0.665 (0.202–2.097)	0.647 (0.202–1.002)	0.607 (0.162–0.992)	0.875
116/89 ratio, median (range)^†^	1.182 (0.754–1.589)	1.245 (0.754–1.589)	1.095 (0.444–1.554)	0.026

	All patients	Healthy donors		

PARP1-mRNA, median (range)^1^	0.094 (0.001–3.490)	0.24 (0.024–1.762)		0.364

^1^PARP1/ACTB ratio; ^2^PARP1/ACTB expression ratio.

∗Correlation between pre- and posttreatment levels was performed using the related samples Wilcoxon Signed Rank test.

^†^Four (4/26) patients did not have a measurable 116 kDa molecule. One of them was in the 15-patient group that was given treatment. Following treatment, 3/15 patients did not have a measurable 116 kDa molecule. For these patients the calculation of 116/89 ratio was not performed, and they were excluded from the relevant statistical analysis.

## References

[1] Krishnakumar R, Kraus WL (2010). The PARP side of the nucleus: molecular actions, physiological outcomes, and clinical targets. *Molecular Cell*.

[2] Carey LA, Sharpless NE (2011). PARP and cancer—if it's broke, don't fix it. *The New England Journal of Medicine*.

[3] Kim MY, Zhang T, Kraus WL (2005). Poly(ADP-ribosyl)ation by PARP-1: ‘PAR-laying’ NAD^+^ into a nuclear signal. *Genes and Development*.

[4] D'Amours D, Desnoyers S, D'Silva I, Poirier GG (1999). Poly(ADP-ribosyl)ation reactions in the regulation of nuclear functions. *Biochemical Journal*.

[5] Oei SL, Shi Y (2001). Transcription factor Yin Yang 1 stimulates poly(ADP-ribosyl)ation and DNA repair. *Biochemical and Biophysical Research Communications*.

[6] Kun E, Kirsten E, Ordahl CP (2002). Coenzymatic activity of randomly broken or intact double-stranded DNAs in auto and histone H1 trans-poly(ADP-ribosylation), catalyzed by poly(ADP-ribose) polymerase (PARP I). *The Journal of Biological Chemistry*.

[7] Ogata N, Ueda K, Kawaichi M, Hayaishi O (1981). Poly(ADP-ribose) synthetase, a main acceptor of poly(ADP-ribose) in isolated nuclei. *The Journal of Biological Chemistry*.

[8] Kraus WL, Lis JT (2003). PARP goes transcription. *Cell*.

[9] Bürkle A (2001). PARP-1: a regulator of genomic stability linked with mammalian longevity. *ChemBioChem*.

[10] Zong WX, Ditsworth D, Bauer DE, Wang ZQ, Thompson CB (2004). Alkylating DNA damage stimulates a regulated form of necrotic cell death. *Genes & Development*.

[11] Ying W, Alano CC, Garnier P, Swanson RA (2005). NAD^+^ as a metabolic link between DNA damage and cell death. *Journal of Neuroscience Research*.

[12] Decker P, Muller S (2002). Modulating poly (ADP-ribose) polymerase activity: potential for the prevention and therapy of pathogenic situations involving DNA damage and oxidative stress. *Current Pharmaceutical Biotechnology*.

[13] Bouchard VJ, Rouleau M, Poirier GG (2003). PARP-1, a determinant of cell survival in response to DNA damage. *Experimental Hematology*.

[14] Kaufmann SH, Desnoyers S, Ottaviano Y, Davidson NE, Poirier GG (1993). Specific proteolytic cleavage of poly(ADP-ribose) polymerase: an early marker of chemotherapy-induced apoptosis. *Cancer Research*.

[15] Soldani C, Scovassi AI (2002). Poly(ADP-ribose) polymerase-1 cleavage during apoptosis: an update. *Apoptosis*.

[16] Edinger AL, Thompson CB (2004). Death by design: apoptosis, necrosis and autophagy. *Current Opinion in Cell Biology*.

[17] Hallek M, Pflug N (2010). Chronic lymphocytic leukemia. *Annals of Oncology*.

[18] Cramer P, Hallek M (2011). Prognostic factors in chronic lymphocytic leukemia-what do we need to know?. *Nature Reviews Clinical Oncology*.

[19] Masood A, Shahshahan MA, Jazirehi AR (2012). Novel approaches to modulate apoptosis resistance: basic and clinical implications in the treatment of chronic lymphocytic leukemia (CLL). *Current Drug Delivery*.

[20] Ouillette P, Fossum S, Parkin B (2010). Aggressive chronic lymphocytic leukemia with elevated genomic complexity is associated with multiple gene defects in the response to DNA double-strand breaks. *Clinical Cancer Research*.

[21] Rosenwald A, Chuang EY, Davis RE (2004). Fludarabine treatment of patients with chronic lymphocytic leukemia induces a p53-dependent gene expression response. *Blood*.

[22] Bacalini MG, Tavolaro S, Peragine N (2012). A subset of chronic lymphocytic leukemia patients display reduced levels of PARP1 expression coupled with a defective irradiation-induced apoptosis. *Experimental Hematology*.

[23] http://www.clinicaltrials.gov/ct2/show/NCT00516724.

[24] http://www.clinicaltrials.gov/ct2/show/NCT00664781.

[25] http://www.clinicaltrials.gov/ct2/show/NCT01945775.

[26] Galluzzi L, Joza N, Tasdemir E (2008). No death without life: vital functions of apoptotic effectors. *Cell Death and Differentiation*.

[27] Cotter TG (2009). Apoptosis and cancer: the genesis of a research field. *Nature Reviews Cancer*.

[28] Chaitanya GV, Alexander JS, Babu PP (2010). PARP-1 cleavage fragments: signatures of cell-death proteases in neurodegeneration. *Cell Communication and Signaling*.

[29] Fischer U, Jänicke RU, Schulze-Osthoff K (2003). Many cuts to ruin: a comprehensive update of caspase substrates. *Cell Death and Differentiation*.

[30] Margolin N, Raybuck SA, Wilson KP (1997). Substrate and inhibitor specificity of interleukin-1*β*-converting enzyme and related caspases. *The Journal of Biological Chemistry*.

[31] Soldani C, Lazzè MC, Bottone MG (2001). Poly(ADP-ribose) polymerase cleavage during apoptosis: when and where?. *Experimental Cell Research*.

[32] D'Amours D, Sallmann FR, Dixit VM, Poirier GG (2001). Gain-of-function of poly(ADP-ribose) polymerase-1 upon cleavage by apoptotic proteases: implications for apoptosis. *Journal of Cell Science*.

[33] Eguchi Y, Shimizu S, Tsujimoto Y (1997). Intracellular ATP levels determine cell death fate by apoptosis or necrosis. *Cancer Research*.

[34] Lemaire C, Andréau K, Souvannavong V, Adam A (1998). Inhibition of caspase activity induces a switch from apoptosis to necrosis. *FEBS Letters*.

